# In Vitro Wound-Healing Potential of Phenolic and Polysaccharide Extracts of *Aloe vera* Gel

**DOI:** 10.3390/jfb15090266

**Published:** 2024-09-13

**Authors:** Andreea Iosageanu, Elena Mihai, Ana-Maria Seciu-Grama, Elena Utoiu, Alexandra Gaspar-Pintiliescu, Florentina Gatea, Anisoara Cimpean, Oana Craciunescu

**Affiliations:** 1 Faculty of Biology, University of Bucharest, 91–95, Splaiul Independentei, 050095 Bucharest, Romania; andreea.iosageanu@incdsb.ro; 2 National Institute of Research and Development for Biological Sciences, 060031 Bucharest, Romania; elena.mihai@incdsb.ro (E.M.); anamaria.seciu@incdsb.ro (A.-M.S.-G.); elena.utoiu@incdsb.ro (E.U.); alexandra.gaspar@incdsb.ro (A.G.-P.); florentina.gatea@incdsb.ro (F.G.); oana.craciunescu@incdsb.ro (O.C.)

**Keywords:** *Aloe vera*, phenolic compounds, polysaccharides, wound healing, antioxidant, anti-inflammatory, antimicrobial

## Abstract

The present study aimed to conduct a comparative investigation of the biological properties of phenolic and polysaccharide extracts obtained using an ultrasound-assisted technique from *Aloe vera* gel and their effects on each stage of the wound healing process in in vitro experimental models. HPLC analysis showed that the phenolic extract contained aloin, ferulic, and caffeic acid, as well as quercetin dihydrate, as major compounds. Capillary zone electrophoresis indicated the prevalence of mannose and glucose in the polysaccharide extract. Cell culture testing revealed the anti-inflammatory properties of the phenolic extract at a concentration of 0.25 mg/mL through significant inhibition of pro-inflammatory cytokines—up to 28% TNF-α and 11% IL-8 secretion—in inflamed THP-1-derived macrophages, while a pro-inflammatory effect was observed at 0.5 mg/mL. The phenolic extract induced 18% stimulation of L929 fibroblast proliferation at a concentration of 0.5 mg/mL, enhanced the cell migration rate by 20%, and increased collagen type I synthesis by 18%. Moreover, the phenolic extract exhibited superior antioxidant properties by scavenging free DPPH (IC_50_ of 2.50 mg/mL) and ABTS (16.47 mM TE/g) radicals, and 46% inhibition of intracellular reactive oxygen species (ROS) production was achieved. The polysaccharide extract demonstrated a greater increase in collagen synthesis up to 25%, as well as antibacterial activity against *Staphylococcus aureus* with a bacteriostatic effect at 25 mg/mL and a bactericidal one at 50 mg/mL. All these findings indicate that the phenolic extract might be more beneficial in formulations intended for the initial phases of wound healing, such as inflammation and proliferation, while the polysaccharide extract could be more suitable for use during the remodeling stage. Moreover, they might be combined with other biomaterials, acting as efficient dressings with anti-inflammatory, antioxidant, and antibacterial properties for rapid recovery of chronic wounds.

## 1. Introduction

The process of skin wound healing is primarily characterized by four distinct yet overlapping phases: hemostasis, inflammation, proliferation, and remodeling. These phases are regulated by pro-/anti-inflammatory cytokines and chemokines that initiate a series of cellular and molecular events involved in cell proliferation, migration, and differentiation, as well as secretion of extracellular matrix components [1]. The physiological process of skin wound healing can be disrupted by local factors, including foreign objects, infections, mechanical stress, or tissue hypoxia, and systemic factors like aging, malnutrition and malabsorption, vascular insufficiency, obesity, or metabolic disorders [2,3], resulting in chronic wounds or the formation of excessive scar tissue [4]. As a result, the complexity of the wound-healing process represents a significant global challenge in terms of development of suitable wound dressings.

In addition to safeguarding the wound against infections, maintaining optimal moisture levels, and facilitating removal of excess exudate [5], an ideal wound dressing should also accelerate the healing process without incurring high costs [6]. One effective approach to achieving this goal involves integrating bioactive molecules isolated from medicinal plants into the wound dressing. These bioactive compounds can stimulate the secretion of growth factors, protect tissue from oxidative stress, and ultimately enhance the healing process for successful wound management [7].

*Aloe vera* L. (*Aloe barbadensis* Miller), the best-known species in the Liliaceae family, is a succulent plant that has been used for centuries in traditional medicine and is now popular as a houseplant and skincare product [8]. *Aloe vera* has green leaves that consist of an outer green rind (epidermis), an inner colorless pulp (parenchyma), and bitter yellow exudate between the epidermis and parenchyma (latex). The part of the plant with medicinal properties is the parenchyma, which contains parenchymatous cells that produce a mucilaginous liquid referred to as *Aloe vera* gel [9]. The composition of *Aloe vera* gel is primarily water (99–99.5%), with 0.5–1% bioactive compounds [10]. These compounds include carbohydrates (monosaccharides such as glucose, mannose, arabinose, and galactose and polysaccharides such as acemannan, hyaluronic acid, and glucomannan), minerals (calcium, magnesium, iron, copper, chromium), proteins (lectins), enzymes (amylase, catalase, cellulase, acid phosphatase, peroxidase, superoxide dismutase), phenolic compounds (aloe-emodin, aloin A and B), salicylic acid, sterols (β-sitosterol, campesterol, cholesterol, lupeol), and vitamins (A, B complex, C, E) [11,12,13]. However, their distribution in aloe gel varies according to the plant part, harvesting season, geographical location, and soil quality and moisture [10].

Different studies have highlighted the impacts of extraction techniques, such as conventional vs. emerging green ones, on the ultimate composition of aloe extracts [14]. In recent years, ultrasound- and microwave-assisted techniques have been more frequently used as green technologies for the isolation of bioactive compounds from several plant sources, including aloe, resulting in higher extraction yields compared to conventional extraction and different phenolics profiles, alongside time reduction, energy-saving effects, and low environmental impact [15].

The bioactive constituents of *Aloe vera* gel studied for their wound healing properties were phenolic compounds [16] and polysaccharides [17]. Phenolic compounds exhibited notable and significantly correlated antioxidant activity [18], mainly due to flavonoids and tannins, which could scavenge free radicals [19], and aloin and aloesin, which could enhance skin photoprotection [20] and reduce inflammation due to sunburns [21,22]. Aloe phenolics modulated the cellular redox status and down-regulated the glycation of antioxidant enzymes through major biochemical pathways, preventing complications from diabetes in rats [19].

In turn, polysaccharides have demonstrated significant immunostimulatory effects [17] by decreasing NLRP3 inflamasome expression and inflammatory cytokine secretion in LPS-stimulated macrophage culture [23], along with inhibition of the cycloxygenase pathway and prostaglandin E2 production [21]. Pain scores can also be diminished by application of 2% *Aloe vera* gel in aphthous stomatitis [24]. Polysaccharides of *Aloe vera* gel have also shown antibacterial activity through a mechanism of stimulation of phagocytic leucocytes, acting on fungi and several bacteria, including *Helicobacter pylori*, suggesting a future application based on synergistic activity along with antibiotics to treat gastric infections [15,25].

It has been demonstrated that *Aloe vera* gel can accelerate wound healing in vitro, mainly by promoting the proliferation and migration of skin cells, fibroblasts, and keratinocytes, in an ex vivo assay [26]. Cutaneous fibrosis and wound contraction were positively modulated by *Aloe vera* gel in full-thickness diabetic wounds in rats [27]. Clinical studies have reported its efficacy in treating various skin conditions, such as dermatitis [28], aphthous stomatitis [24], postoperative wounds [29], and first- and second-degree burns [30], in which topical administration improves the re-epithelialization rate and wound healing duration compared to silver sulfadiazine [31]. Still, the reported results are controversial and the mechanisms of action remain unclear.

In this context, the aim of this study was to isolate phenolic and polysaccharide preparations from *Aloe vera* gel using the green technology of ultrasound-assisted extraction (UAE) and, for the first time, to comparatively investigate their effects on each phase of the wound-healing process using experimental models in vitro to assess the inflammation, fibroblast proliferation and migration, and collagen synthesis. In addition, their antioxidant and antibacterial properties were analyzed to delineate the medicinal advantages of each extract for further limited applied formulations in certain stages of healing.

## 2. Materials and Methods

### 2.1. Materials

*Aloe vera* leaves were sourced from Botanical Garden “D. Brandza”, University of Bucharest, in 2022. The leaves were harvested in the morning from plants which were at least 3 years old, as previously recommended for maximizing the content of bioactive compounds [32]. They had an average mass of 211 g, length of 55 cm, base width of 6 cm, and base thickness of 1.3 cm, and an average volume of 112.25 cm^3^ was calculated, taking into account their conical shape.

The murine NCTC clone L929 fibroblast cell line was purchased from ECACC (Sigma-Aldrich, Taufkirchen, Germany). The human THP-1 monocytic leukemia cell line was purchased from ATCC (Sigma-Aldrich, Germany). Minimum Essential Medium (MEM), Roswell Park Memorial Institute 1640 (RPMI 1640) medium, fetal bovine serum (FBS), penicillin–streptomycin–neomycin antibiotic mixture (PSN), and other chemical reagents of analytical purity were purchased from Sigma-Aldrich (Germany), unless otherwise specified.

### 2.2. Preparation of Extracts

The leaves of *Aloe vera* were cut at the base of the plant, and then they were left upright for 30 min to drain the latex [33]. Then, the leaves were washed in distilled water and the top, spines, and peel were separated from the inner fillets using a scalpel. The fillets were homogenized using a blender, and the obtained gel was lyophilized.

Then, the phenolic extract was prepared from freeze-dried aloe gel dissolved in 70% (*v*/*v*) ethanol solution at a ratio of 1:100 (*w*/*v*). The solution underwent UAE in a sonication bath (ELMA, Pforzheim, Germany) at high intensity and low frequency (60 kHz, 550 W) to disrupt cell walls and membranes [34]. This lasted for 20 min, with cycles of 5 min on and 2 min off to maintain a temperature of 25 °C. The resulting solution was then centrifuged at 5000× *g* and 4 °C for 30 min, and the supernatant containing the phenolic extract was collected, evaporated at 40 °C, and freeze-dried. The resulting solid mass was packed and stored in a dry place.

The polysaccharide extract was prepared according to the previous UAE method with minor modifications [35]. Freeze-dried *Aloe vera* gel was dissolved in ultrapure water at a ratio of 1:100 (*w*/*v*), with stirring at 25 °C, for 2 h. Subsequently, the solution underwent sonication at high intensity and low frequency (60 kHz, 550 W) for 20 min at 25 °C, with cycles of 5 min on and 2 min off. The resulting solution was then centrifuged at 5000× *g* and 4 °C for 30 min. The supernatant containing water-soluble polysaccharides was pipetted into a triple volume of cold absolute ethanol, leading to immediate precipitation of polysaccharides. The solution was stirred continuously for 45 min at 25 °C, then incubated overnight at 4 °C in a sealed container. After centrifugation at 5000× *g* and 4 °C for 30 min, the obtained pellet was freeze-dried and stored in a dry place.

**Figure 1:**
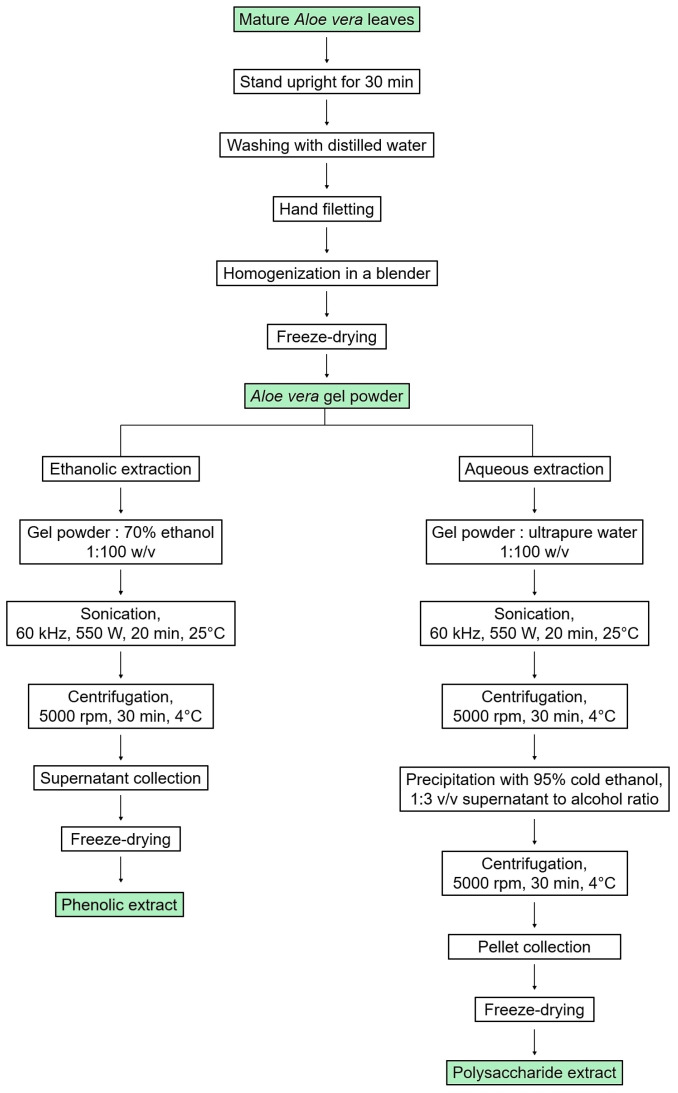
Scheme of the preparation of phenolic and polysaccharide extracts from *Aloe vera* gel using UAE.

A schematic representation of the extraction process is given in Figure 1.

The extraction yield (%) was calculated according to the following equation:

(1) $$Extraction\;yield\;\left(\%\right)=\frac{W_{2}}{W_{1}}\times 100$$

where W_1_ is the dry weight of initial aloe gel powder and W_2_ is the dry weight of each extract.

### 2.3. Determination of Total Phenolic, Flavonoid, and Carbohydrate Content

The total phenolic content was determined using the Folin–Ciocalteu method as previously described [36], with minor modifications. Briefly, 150 μL of extract (10 mg/mL) was mixed with 750 μL of Folin–Ciocalteu reagent (2 N) and incubated at 25 °C for 5 min. The mixture was further incubated with 2 mL of 12% sodium carbonate in the dark at 25 °C for 30 min. At the end, 13 mL of distilled water was added, and the absorbance (Abs) was measured at a wavelength of 765 nm with a V-650 UV-VIS spectrophotometer (JASCO, Tokyo, Japan). A series of different concentrations of gallic acid in the range of 0–500 µg/mL were used to build the calibration curve.

The total flavonoid content was determined according to an aluminum chloride assay [37]. Briefly, 0.5 mL of extract (25 mg/mL) was mixed with 1.5 mL methanol, 0.1 mL of 10% aluminum chloride, 0.1 mL of 1 M sodium acetate, and 2.8 mL of distilled water. Then, the mixture was incubated at 25 °C for 30 min. The Abs was measured at a wavelength of 510 nm with a V650 UV/VIS spectrophotometer (JASCO, Japan). A series of different concentrations of quercetin in the range of 0–100 µg/mL were used to build the calibration curve.

The total carbohydrate content was determined according to the phenol–sulfuric acid method with slight modifications [38]. Thus, 50 μL of extract (0.5 mg/mL) was mixed with 150 μL of conc. H_2_SO_4_ and 30 μL of 5% aqueous phenol. The mixture was heated at 95 °C for 10 min. After cooling, the Abs was read at a wavelength of 490 nm with a Sunrise microplate reader (Tecan, Salzburg, Austria). A series of different concentrations of glucose in the range of 0–500 µg/mL were used to build the calibration curve.

### 2.4. High-Performance Liquid Chromatography (HPLC) Analysis

Phenolic compounds were identified and quantified with a 1200 Infinity HPLC system (Agilent Technologies, Santa Clara, CA, USA) consisting of a quaternary pump, a reverse-phase column C18 Zorbax Eclipse XDB (4.6 i.d. × 150 mm) with a 5 µm particle size, and a diode array detector (DAD). The sample was prepared by solving in 80% methanol (4 mg/mL) and filtration through 0.45 μm membranes. The volume injected into the column was 10 μL, and the working temperature of the column was 27 °C. A step gradient of the mobile phase was used for elution as follows: 2–20% acetonitrile (solvent B) in phosphoric acid in water, pH 2.12 (solvent A) for 30 min; 20–30% B in A for 10 min; 30% B in A for 10 min; 30–2% B in A for 10 min; and 2% B in A for 15 min [39] at a flow rate of 0.7 mL/min. The Abs was read at 280 and 320 nm.

The aloin content of the extracts was determined using the same system, operated at 35 °C, with a gradient of the mobile phase as follows: 20–35% acetonitrile (solvent B) in acetic acid in water (solvent A), 13 min; 35–98% B in A, 17 min; 98–20% B in A, 1 min; and 20% B in A, 9 min [40], at a flow rate of 1 mL/min. The Abs was read at 357 nm.

For quantification of the main phenolic acids, flavonoids, and aloin, the peak area integration was conducted based on the standard calibration curves and Chemstation B.04.03-SP1 software.

### 2.5. Capillary Zone Electrophoresis

The monosaccharide composition of the polysaccharide extract was analyzed by capillary zone electrophoresis on a CE Agilent instrument equipped with a fused-silica column (0.05 i.d. × 720 mm) (Agilent Technologies, Waldbronn, Germany) and DAD. The sample (20 mg) was hydrolyzed in 4 M tri-fluoroacetic acid (2 mL) at 120 °C for 6 h, neutralized with 0.3 M NaOH solution, and subsequently derivatized with 1-phenyl-3-methyl-5-pyrazolone. The separation was carried out in 90 mM boric acid solution at pH 9.8 at 30 °C, applying a voltage of 30 kV [41]. The Abs was read at 245 nm. Data analysis was performed using ChemStation software.

### 2.6. Cell Culture Testing

#### 2.6.1. Determination of Inflammatory Cytokines Production by ELISA

An in vitro experimental model of inflammation was developed according to Craciunescu et al. [42]. Briefly, THP-1 cells differentiated to macrophages in 24-well plates were inflamed by treatment with 10 ng/mL lipopolysaccharide (LPS) in the presence of cytocompatible concentrations of extracts for 24 h. Then, the culture media were centrifuged at 1000× *g* for 10 min. TNF-α and IL-8 production were determined in supernatants using specific ELISA kits (R&D Systems Inc., Minneapolis, MN, USA). The Abs was measured at a wavelength of 450 nm with a Spectrostar Nano microplate reader (BMG Labtech, Ortenberg, Germany). Untreated cells served as negative controls. The results were normalized to cell viability values, which were determined under standard conditions of cultivation by Neutral Red uptake test [43]. Briefly, THP-1-derived macrophages were treated with different concentrations of extracts (0.1–3 mg/mL) for 24 h. Then, the medium was replaced with Neutral Red reagent (50 μg/mL) prepared in fresh medium, and the plates were further incubated for 3 h. After cell washing in phosphate-buffered saline (PBS), cells were fixed for 3 min and decolorized for 15 min on a shaker. The Abs was read at a wavelength of 540 nm with a Sunrise microplate reader (Tecan, Austria). The results were calculated using the following equation:

(2) $$Cell\;viability\;\left(\%\right)=\frac{{Abs}_{sample}}{{Abs}_{control}}\times 100$$

#### 2.6.2. Determination of Cell Viability by Neutral Red Assay and Morphology Observations

L929 fibroblast cells were seeded at a density of 4 × 10^4^ cells/mL in 96-well plates and cultured in MEM overnight. Then, the culture medium was replaced with fresh medium containing different concentrations of *Aloe vera* extracts (0.1–3 mg/mL), and the plates were incubated for 24 and 48 h. The viability of the cells was determined according to Neutral Red uptake test, as described in Section 2.6.1. The Abs values were directly proportional to the number of viable cells.

For cell morphology observations, L929 cells seeded at a density of 4 × 10^4^ cells/mL in 24-well plates were treated as described above for cell viability experiments. After 48 h of incubation in standard conditions, the cells were imaged using an AxioStar Plus inverted microscope (Carl Zeiss, Jena, Germany).

#### 2.6.3. In Vitro Assessment of Cell Migration

Cell migration was assayed by an in vitro scratch assay on L929 cells, as previously described [44]. Briefly, cells were seeded at a density of 3 × 10^5^ cells/mL in 24-well plates, which were incubated in standard conditions overnight. Then, the monolayers were scratched with a 200 μL micropipette tip and washed with PBS in order to remove cellular debris. Next, the cells were cultivated in the presence of different concentrations of extracts and the plates were incubated for 24 h. Images of the monolayer were taken at an AxioStar Plus inverted microscope equipped with a digital camera (Carl Zeiss, Germany). ImageJ 1.53a software was used to analyze the images. Untreated cells served as negative controls. The rate of cell migration was calculated according to the following formula:

(3) $$Cell\;migration\;rate\;\left(\%\right)=\frac{{Area}_{t0}-{Area}_{t24}}{{Area}_{t0}}\times 100$$

where Area_t0_ is the area value at t = 0 h and Area_t24_ is the area value at t = 24 h.

#### 2.6.4. Determination of Collagen Type I Production by ELISA

The in vitro experimental model for the determination of collagen production was developed according to Tewtrakul et al. [45] with minor modifications. Briefly, the cells were seeded at a density of 1 × 10^5^ cells/mL in 24-well plates, which were incubated in standard conditions for 24 h. Then, the culture medium was replaced with serum-free medium containing different concentrations of extract and the plates were further incubated for 72 h. Subsequently, the culture media were centrifuged at 1000× *g* for 10 min, and the supernatants were analyzed for production of collagen type I using a specific competitive ELISA kit (MyBioSource, San Diego, CA, USA). The Abs was read at a wavelength of 450 nm using a Spectrostar Nano microplate reader (BMG Labtech, Germany). The results were standardized based on cell viability measurements. Untreated cells served as negative controls.

### 2.7. Determination of Antioxidant Activity

#### 2.7.1. Determination of Free Radical Scavenging Capacity

The free radical scavenging capacity of phenolic and polysaccharide extracts was determined by 2,2-Diphenyl-1-picrylhydrazyl (DPPH) and 2,2′-azino-bis(3-ethylbenzothiazoline-6-sulfonic acid (ABTS) assays. The DPPH assay evaluates the ability of stable free DPPH radicals to decolorize in the presence of antioxidants. The scavenging capacity of *Aloe vera* extracts was analyzed according to Craciunescu et al. [46] with minor modifications. After incubation of 0.25–4 mg/mL extract concentrations (150 µL) with 0.25 mM DPPH solution (1.35 mL) and 0.1 M Tris-HCl buffer with a pH of 7.4 (0.9 mL) at 25 °C in the dark for 30 min, the Abs was read at a wavelength of 517 nm using a V650 UV-VIS spectrophotometer (JASCO, Japan). The control was prepared by sample replacement with buffer. The scavenging capacity was calculated according to the following equation:

(4) $$Free\;DPPH\;radicals\;scavenging\left(\%\right)=\frac{{Abs}_{control}-{Abs}_{sample}}{{Abs}_{control}}\times 100$$

The inhibitory concentration IC_50_ was determined as the extract concentration that inhibited 50% of free DPPH radicals.

The Trolox equivalent antioxidant capacity (TEAC) assay evaluated the ability of antioxidants to scavenge free ABTS radical cations. The scavenging capacity of *Aloe vera* extracts was determined according to Gaspar-Pintiliescu et al. [47], with some modifications. Briefly, the stock solution containing free ABTS radical cations, which was generated by incubation of 7 mM ABTS solution with 2.45 mM potassium persulfate in a 1:1 (*v*/*v*) ratio at 25 °C in the dark for 16 h, was diluted with distilled water until the Abs read at 734 nm was 0.70 ± 0.02. After mixing 10 mg/mL extract (100 µL) with ABTS reagent (1 mL) and incubation at 25 °C in the dark for 10 min, the Abs was read at 734 nm using a V650 UV-VIS spectrophotometer (JASCO, Japan). Different concentrations of Trolox (0–150 µM), a known antioxidant agent, served to build a standard curve.

#### 2.7.2. Quantification of Intracellular Reactive Oxygen Species (ROS) Production

An experimental in vitro model of oxidative stress was developed in L929 cells to assess the antioxidant activity of aloe extract by determination of the intracellular ROS production, as previously described [48]. The cells were seeded at a density of 5 × 10^4^ cells/mL in 12-well plates, then incubated in standard conditions for 24 h. Then, the culture medium was replaced with fresh medium containing different concentrations of phenolic and polysaccharide extracts, and the plate was further incubated for 48 h. Subsequently, the cells were incubated with 50 µM t-butyl hydroperoxide (t-BHP) for 30 min and 2′,7′-dichlorofluorescein diacetate (DCFH-DA) (10 µM) for 30 min. The fluorescence signals obtained after the dye reaction with ROS and formation of DCF were measured by flow cytometry using LSR II equipment (Becton Dickinson, Franklin Lakes, NJ, USA). The obtained histograms were analyzed using FACSDiva V6.1.3 and FlowJo V10 software to assess the inhibition of intracellular ROS production. Cells treated with ascorbic acid (12 µM) served as an antioxidant control.

### 2.8. Determination of Antibacterial Activity

The microdilution test was performed to assess the antibacterial properties of phenolic and polysaccharide UAE extracts of Aloe vera gel against Gram-positive *Staphylococcus aureus* (ATCC 25923) and Gram-negative *Pseudomonas aeruginosa* (ATCC 27853). The choice of the microdilution method over the agar diffusion test was deliberate, as polysaccharides have limited diffusion capabilities in agar [49]. Bacterial strains were cultivated in de Man, Rogosa, and Sharpe (MRS) nutrient medium and Luria–Bertani (LB) nutrient medium, followed by aerobic incubation at 37 °C. Each well of a 96-well plate was initially filled with 50 µL of growth medium, followed by 50 µL of serial dilutions of the sample, starting from 100 mg/mL stock solution. Subsequently, 50 µL of bacterial suspension with a turbidity adjusted to 0.5 μL McFarland was added, and the plates were incubated at 37 °C for 24 h. The Abs was measured at 600 nm with a Sunrise microplate reader (Tecan, Austria), and the values were proportional to the bacterial growth. The minimum inhibitory concentration (MIC) was determined as the lowest concentration of extract that effectively inhibited the microbial growth after 24 h of incubation. Then, the concentration representing MIC and higher concentrations of the extract were plated on agar plates and incubated overnight. The growth of bacterial colonies on the agar was observed, and the lowest concentration at which no bacterial growth was observed represented the Minimum Bactericidal Concentration (MBC).

### 2.9. Statistical Analysis

All the experiments were conducted in triplicate, and the results are expressed as mean ± standard deviation (SD) (n = 3). Statistically significant differences between control and sample groups were determined by two-tailed, two-sample, equal-variance Student’s *t*-tests using Microsoft Office Excel 2019. Differences were considered statistically significant at *p* < 0.05.

## 3. Results and Discussion

### 3.1. Preparation of Aloe vera Extracts and Yield

In order to better preserve the natural composition and properties of the *Aloe vera* gel without the need of preservatives, the fresh gel was lyophilized. The calculated moisture content of the fresh gel was high, with a value of 99.03%, confirming previous data [10]. The process of ethanol extraction of *Aloe vera* gel by UAE had a calculated yield of 44.02%, while water extraction by UAE followed by ethanol precipitation reached a value of 52.14% (Table 1).

### 3.2. Total Phenolic, Flavonoid, and Carbohydrate Content

The results are presented in Table 1. The data showed a high phenolic content of 33.25 mg GAE/g and a total flavonoid content of 1.96 mg QE/g in the ethanolic extract, while the aqueous extract had a low phenolic content (0.83 mg GAE/g) and no flavonoids were detected. With regard to the total carbohydrate content, the water extract presented a high value (936.95 mg GE/g), while the ethanol extract had a value of 644.65 mg GE/g. Polysaccharides are the most abundant type of compounds in *Aloe vera* gel and they are easily extracted in aqueous environments, followed by ethanol precipitation [50]. The relative high content of total carbohydrates in the phenolic extract could be a result of the presence of glycosides [51].

Our findings were consistent with previous studies reporting values of 28.44 mg GAE/g [52] and 30 mg GAE/g [53] for the total phenolic content in ethanol extract of *Aloe vera* gel. Mehmood et al. [54] reported a total phenolic content of 17.13 mg GAE/g in the ethanol extract of *Aloe vera* gel, a slightly lower value compared to our study. In a study utilizing UAE with 70% ethanol as a solvent, the total phenolic content in the aloe rind by-product was measured at 9.24 mg GAE/g, while with water as the solvent, the result was 5.22 mg GAE/g [55].

In terms of total flavonoid content, it was previously reported that UAE in ethanol applied to *Aloe vera* gel yielded a quantity of 3.37 mg QE/g, indicating a higher flavonoid content in comparison to the present study, while Soxhlet extraction using ethanol resulted in a quantity of 0.29 mg QE/g, notably lower compared to that obtained in the present research [56]. Ray et al. [57] observed variations in the flavonoid contents in *Aloe vera* gel based on plant age, with three-year-old plants showing the highest content at 29.75 mg rutin equivalents (RE)/g, followed by two-year-old and four-year-old plants at 18.63 mg RE/g and 11.00 mg RE/g, respectively. In another study, various extracts of *Aloe vera* leaves obtained using different solvents were compared, revealing variations in the flavonoid content among them. The ethanol extract displayed the highest flavonoid content at 0.75 RE mg/g, followed by the methanol extract at 0.5 mg RE/g, while the acidic methanol extract showed no detectable flavonoids [36]. Another quantitative investigation reported that the ethanol extract of *Aloe vera* leaves contained 54.95 mg QE/g of flavonoids, while the methanol extract contained 73.26 mg QE/g, contradicting previous findings that suggested that the flavonoid content in ethanol extraction is higher compared to methanol extraction [58]. It is noteworthy that in the study presented by Hossen et al. [36], the ethanol extract also contained water, potentially aiding in the extraction of flavonoids. The variation in total phenolic and flavonoid content observed among different studies highlighted the impacts of extraction techniques and plant sources on the ultimate composition of the extracts.

### 3.3. Phenolics and Monosaccharides Profile

The amounts of identified phenolic compounds are presented in Table 2. The anthraquinone derivatives aloin B and aloin A were found in the phenolic extract of *Aloe vera*, accounting for 3.777 and 5.158 mg/g, respectively. The most abundant phenolic acids were ferulic acid (0.328 mg/g), caffeic acid (0.316 mg/g), and chlorogenic acid (0.285 mg/g). The most abundant flavonoids were quercetin dihydrate (0.351 mg/g), rutin trihydrate (0.308 mg/g), and myricetin (0.298 mg/g).

In a previous study conducted by Anibarro-Ortega et al. [59], lower concentrations of aloin B (0.24 mg/g) and aloin A (1.24 mg/g) were found in the ethanolic extract of *Aloe vera* fillet, while higher concentrations of aloin B (13.54 mg/g) and aloin A (23.44 mg/g) were found in the ethanolic extract of *Aloe vera* mucilage. In the present research, both the fillet of the innermost layer and the mucilage were considered as *Aloe vera* gel and served as the basis for the extracts. Therefore, depending on the proportion of fillet to mucilage, the quantity of anthraquinones in *Aloe vera* extracts might vary.

Fedoul et al. [60] reported that the water and ethanol extracts of *Aloe vera* gel contained chlorogenic acid at concentrations of 0.20 mg/g and 0.14 mg/g, respectively, which are lower values compared with the present study (0.285 mg/g). Sumi et al. [61] found comparable levels of ferulic acid, caffeic acid, and rutin in the ethanolic extract of whole *Aloe vera* leaves, with concentrations of 0.412 mg/g, 0.230 mg/g, and 0.396 mg/g, respectively. In a study presented by Yahya et al. [25], the most abundant phenolic compound found in *Aloe vera* gel was chlorogenic acid, followed by pyrocatechol and catechin.

The differences in phenolic profile and quantity likely arose from the plant part and the extraction method. Thus, it was previously reported that the most abundant phenolic compound in *Aloe vera* leaf skin was sinapic acid, with a concentration of 0.54 mg/g, while that in aloe flowers was gentisic acid, with a concentration of 1.01 mg/g [62]. Chlorogenic acid, ferulic acid, and myricetin were also detected, but in lower concentrations than in the present study.

The monosaccharide profile and their contents in the polysaccharide extract of *Aloe vera* gel are presented in Table 3. The analysis reveals that the predominant monosaccharide in the sample was mannose, with a concentration of 534.273 mg/g, followed by glucose at 313.881 mg/g. Additionally, galactose and glucuronic acid were identified in amounts of 16.680 mg/g and 11.092 mg/g, respectively. Thus, the composition of the polysaccharide extract, expressed as percentages, was 61% mannose, 36% glucose, 1.9% galactose, and 1.3% glucuronic acid.

This composition indicated the presence of acemannan, the main bioactive polysaccharide of *Aloe vera* gel, which could play a crucial role in the therapeutic properties associated with wound healing [12]. Acemannan is predominantly composed of mannose (84.9%), glucose (7.2%), and galactose (3.9%) [63]. A similar study revealed a composition of 63% mannose, 12% glucose, and 0.6% galactose for the polysaccharide extract isolated from fresh *Aloe vera* gel obtained from 3-year-old plants using water extraction, as well as the presence of acemannan, along with other polysaccharides [64]. Trehalose, a disaccharide composed of glucose molecules linked by α-glycosidic bonds, was only identified in *Aloe vera* flowers, at low concentrations between 0.10 and 0.34 mg/g [65].

### 3.4. Effect on the Inflammatory Phase of Wound Healing

**Figure 2:**
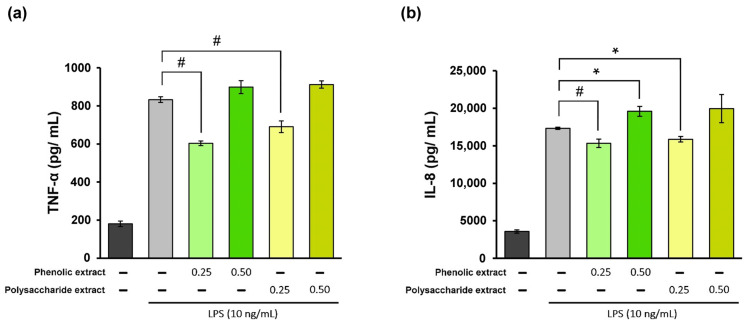
Quantification of TNF-α (**a**) and IL-8 (**b**) levels in LPS-stimulated THP-1-derived macrophages (light grey) and after treatment with phenolic and polysaccharide extract of *Aloe vera* gel. Untreated cells served as the control group (dark grey). * *p* < 0.05, # *p* < 0.01.

The inflammatory phase of the wound-healing process is essential for promoting tissue repair. However, excessive inflammatory cytokines can lead to tissue damage. Therefore, it can be stated that the utilization of products possessing anti-inflammatory properties may prove to be advantageous in accelerating the healing process [66]. In the present study, treatment of THP-1-derived macrophages with different concentrations of *Aloe vera* extracts showed high cytocompatibility at 0.25 and 0.5 mg/mL, and these values were selected for inflammation experiments. In the experimental model of LPS-induced inflammation in THP-1-derived macrophages treated with *Aloe vera* extracts, the levels of TNF-α and IL-8 pro-inflammatory cytokine secretion were quantified directly in the culture medium using a specific ELISA kit. The results are presented in Figure 2. The data showed that stimulation of human THP-1-derived macrophages with LPS resulted in 360% increase in TNF-α secretion compared to the negative control, as well as a 385% increase in IL-8 secretion. Treatment with *Aloe vera* phenolic and polysaccharide extracts led to a significant (*p* < 0.01) reduction in TNF-α secretion at a concentration of 0.25 mg/mL (Figure 2a). The phenolic extract has induced a 28% decrease in the TNF-α level below the value in the positive control, while the polysaccharide extract treatment resulted in a 17% reduction in the TNF-α level. However, an increase in the TNF-α level was found at a concentration of 0.5 mg/mL for both extracts, indicating a potential pro-inflammatory effect.

In terms of IL-8 secretion, the concentration of 0.25 mg/mL phenolic extract provided an 11% decrease in the cytokine level below that in the positive control (Figure 2b), whereas treatment with the polysaccharide extract at the same concentration showed an 8% reduction in the cytokine level. At a concentration of 0.5 mg/mL, both extracts caused increases in IL-8 secretion compared to the positive control, suggesting a pro-inflammatory effect. Therefore, these *Aloe vera* extracts could exhibit and modulate anti-inflammatory/pro-inflammatory properties in the cellular environment which are biomimetic to wound healing. The results also indicated that higher concentrations might not be advantageous for wound healing in vitro.

A similar study conducted in THP-1-derived macrophages revealed that fresh *Aloe vera* gel significantly reduced the LPS-induced secretion of TNF-α and IL-8 pro-inflammatory cytokines [23]. Thus, the treatment with 10% (*v*/*v*) fresh *Aloe vera* gel resulted in an approximately 60% decrease in TNF-α level in LPS-stimulated cells compared to the positive control, while the level of IL-8 decreased by approximately 30%. Consistent with the results in the present study, fresh *Aloe vera* gel demonstrated an inhibitory effect on TNF-α at a greater extent in comparison to IL-8, as well as robust anti-inflammatory activity. However, this study tested a commercially available product stabilized with antioxidants and preservatives that might have played a synergistic effect.

Among aloe compounds, aloin was proved to attenuate inflammation in murine macrophages via inhibition of the JAK1-STAT1/3 signaling pathway and the nuclear translocation of the transcription factors STAT1/3 [67]. Moreover, the modulation of pro-inflammatory cytokines expression was induced by aloin through IkBα and NF-kB p65 phosphorylation decreases in inflamed murine macrophages [68]. A dose-dependent protective effect of aloesone on M1 polarization and apoptosis inhibition has previously been demonstrated in inflammation-stimulated macrophages through inhibition of mTOR, p-mTOR, and HIF-1α, but also of the TLR4 protein [69]. Inflammation was also controlled by acemannan, the polysaccharide in aloe, which promoted the generation of nitric oxide, a key regulator of macrophage functionality, through the terminal mannose receptor [70].

### 3.5. Effect on the Proliferation Phase of Wound Healing

**Figure 3:**
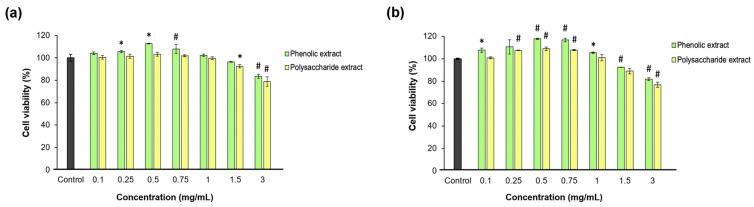
Viability of L929 fibroblasts at 24 (**a**) and 48 (**b**) h of treatment with phenolic and polysaccharide UAE extracts of *Aloe vera* gel, determined by Neutral Red assay. * *p* < 0.05, # *p* < 0.01.

Fibroblast proliferation and migration are extremely important during the proliferation phase of wound healing [71]. In the present study, the effect of each *Aloe vera* extract on L929 fibroblast viability, proliferation, morphology, and migration was investigated after direct contact cultivation. The Neutral Red assay data indicated that neither *Aloe vera* gel extract was cytotoxic at all tested concentrations between 0.1 and 3 mg/mL at either time point, except for 3 mg/mL polysaccharide extract, which induced an insignificant (*p* > 0.05) decrease below the 80% cell viability threshold (Figure 3). Significantly (*p* < 0.05) higher values of cell viability were observed up to 0.75 mg/mL aloe extract, increasing in a dose-dependent manner compared to the untreated control. Higher concentrations induced decreases in cell viability down to 77% for the polysaccharide extract at 48 h of cultivation.

The data also showed that the phenolic and polysaccharide extracts had the highest potency in terms of promoting cell proliferation at concentrations between 0.25 and 0.75 mg/mL compared to the control group at 48 h of cultivation. Thus, at a concentration of 0.5 mg/mL, the phenolic extract induced an 18% increase in L929 fibroblast proliferation, while the polysaccharide extract only induced a 9% increase, but both values were significantly (*p* < 0.05) higher compared to the control group. At extract concentrations higher than 1 mg/mL, the cell proliferation decreased in a dose-dependent manner. These results indicated effective concentrations of aloe extracts at 0.25 and 0.5 mg/mL in terms of a positive relationship with the cell metabolism for further use in experimental models developed in fibroblast-like cells. Lower concentrations had similar responses to untreated cells, while higher concentrations induced interferences in cell metabolism, which should be avoided in vitro in order not to amplify the inflammatory or oxidative stress.

A previous study on L929 fibroblasts revealed that fresh *Aloe vera* gel exhibited good biocompatibility and cell viability values, which reached 150% at concentrations below 250 mg/mL after 24 h and were below 62.5 mg/mL after 48 h of cultivation [72]. The higher biocompatibility threshold observed in this study could be attributed to the use of fresh *Aloe vera* gel, whereas the lyophilized *Aloe vera* gel extracts used in the present study were more concentrated in compounds. In a study on NIH 3T3 fibroblasts, treatment with ethanol extract of *Aloe vera* leaves enhanced fibroblast viability, which is consistent with our findings [73]. The greatest increase in cell viability was noted at a concentration of 5 μg/mL, although all tested concentrations up to 500 μg/mL were biocompatible at 24 h. Interestingly, at 48 h, the cell viability significantly decreased to values between 70% and 50% in a dose-dependent manner.

*Aloe vera* gel can also be incorporated into biomaterial compositions. Silva et al. [74] combined *Aloe vera* gel with gellan gum to create matrices that were subsequently lyophilized, resulting in *Aloe vera* gel sponges. Following testing on L929 cells, it was determined that there was no cytotoxic effect on L929 fibroblasts. The cell viability for cells treated with the biomaterial was 155%, while the viability for cells treated with *Aloe vera* gel alone was 166%. Despite the decrease of 11% in cell viability, it was evident that the beneficial properties of *Aloe vera* gel could be maintained even when the extract was embedded in biomaterial compositions.

**Figure 4:**
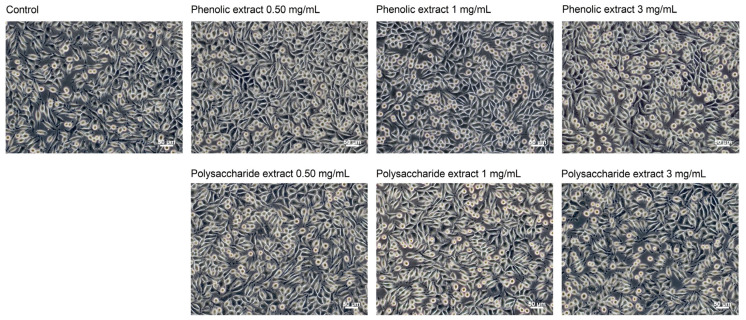
Phase contrast images showing the cell morphology of L929 fibroblasts at 48 h of treatment with phenolic and polysaccharide UAE extracts of *Aloe vera* gel. Scale bar = 50 μm.

Following the morphological observations conducted in the present study, no alterations were detected in cells cultivated in the presence of *Aloe vera* extracts (Figure 4). Fibroblasts in both control and extract-treated cultures displayed characteristic spindle-shaped morphology, which is typical for the fibroblast-like cells. Even at a concentration of 3 mg/mL, the cellular morphology remained unchanged, indicating high cellular viability. Previous studies have shown that the characteristic polysaccharide of aloe, acemannan, could act as a mitogenic stimulator, promoting fibroblast proliferation in skin wound healing through the activation of expression of cyclins D1 and D2, which act as key mediators of cell cycle progression via AKT/mTOR signaling pathway [75].

**Figure 5:**
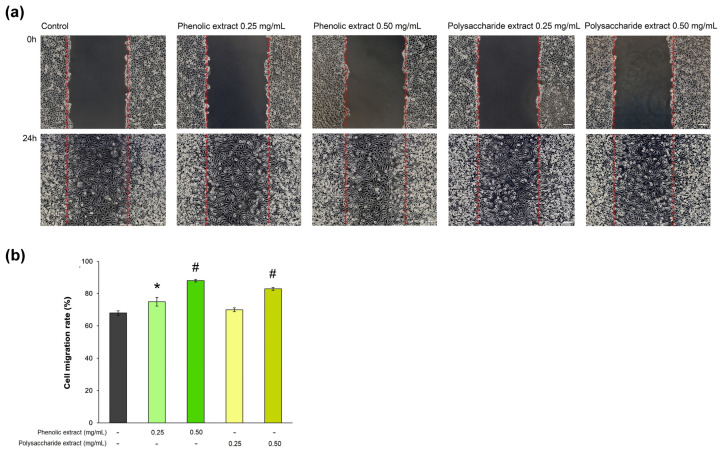
(**a**) Phase contrast images showing cell migration of L929 cells treated with phenolic and polysaccharide UAE extracts of *Aloe vera* gel for 24 h in a scratch model assay. Scale bar = 100 µm; (**b**) cell migration rate determined by ImageJ analysis. * *p* < 0.05, # *p* < 0.01.

In the present study, the rate of L929 fibroblast cell migration was assessed in a wound-repair model observed in vitro during 24 h of treatment with *Aloe vera* extracts. The results are shown in Figure 5. The data indicated that, in the presence of 0.5 mg/mL phenolic extract, the cell migration rate reached 88%, being significantly (*p* < 0.01) increased compared to that of the untreated control (68%). Consequently, the phenolic extract exhibited the greatest effect on the cell migration rate. The percentage of cell migration in cells treated with 0.25 mg/mL phenolic extract was 75%. In the case of the polysaccharide extract, the concentration of 0.5 mg/mL resulted in an 83% cell migration rate within 24 h, while the 0.25 mg/mL concentration treatment reached a value of 70%, close to that in the untreated control.

Accordingly, an in vitro study revealed that *Aloe vera* gel stimulated the proliferation and migration of both fibroblasts and keratinocytes, which are crucial processes in tissue regeneration [26]. In addition, Shafaie et al. [76] reported that *Aloe vera* gel promoted wound healing due to fibroblast proliferation and migration, mediated by the expression of α1β1 integrins playing a central role in cell adhesion and modulating cell signaling pathways mediated by transmembrane protein kinases [77]. In vivo investigations have demonstrated a positive impact of topical and oral administration of *Aloe vera* gel, which effectively accelerated skin healing in mice [78].

Aloin was found to promote the rate of wound closure in vitro and in vivo by increasing chemotaxis, epidermal growth factor expression, and neovascularization [79]. Aloesin also increased cell migration in cultured skin cells through Cdc42 and Rac1 phosphorylation and the wound closure rate in hairless mice through activation of the Smad and MAPK signaling proteins [80].

### 3.6. Effect on the Remodeling Phase of Wound Healing

**Figure 6:**
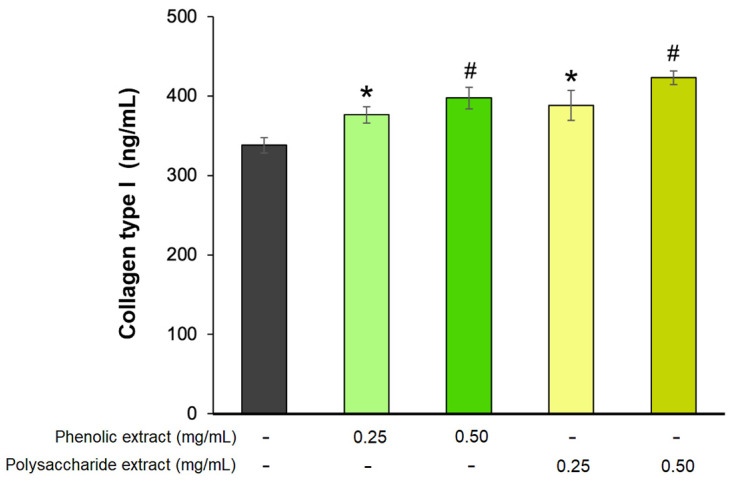
Collagen type I production in L929 fibroblasts after 72 h of treatment with phenolic and polysaccharide UAE extracts of *Aloe vera* gel, as determined by ELISA. * *p* < 0.05, # *p* < 0.01.

The last phase of the wound-healing process, known as the remodeling phase, is a prolonged process that can extend over several years [81]. During this phase, type III collagen is replaced by type I collagen, playing a crucial role in wound contraction and resulting in enhanced tissue strength and structural integrity, thus improving the wound-healing process [82]. In the present study, the effect of *Aloe vera* phenolic and polysaccharide extracts on collagen type I production was analyzed in a fibroblast experimental model. The results showed that the extracts stimulated the secretion of collagen type I in a dose-dependent manner (Figure 6). The highest effect was exhibited by 0.5 mg/mL polysaccharide extract, leading to a 25% increase in collagen secretion, while at 0.25 mg/mL, the treated cells secreted 15% more type I collagen compared to untreated cells. Similarly, a concentration of 0.5 mg/mL phenolic extract increased the collagen synthesis by 18%, while at 0.25 mg/mL, the increase was 11% compared to the untreated control.

These results were in accordance with a previous study highlighting that the polysaccharide extract of *Aloe vera* gel enhanced collagen synthesis in fibroblasts [83]. When conditioned in liposomes, *Aloe vera* gel significantly increased collagen synthesis by 23% in NB1RGB fibroblast cells compared to the untreated cells [84]. In addition, in vivo research indicated that *Aloe vera* gel could enhance wound healing in animal models by promoting collagen synthesis, but also collagen cross-linking and composition [85]. Glucomannan rich in mannose increased the production of collagen as a result of interactions with fibroblast growth factor receptors and fibroblast proliferation [86]. Acemannan gel proved to be most useful in sheep wounds which developed abundant exudate and granulation tissue, acting through mechanisms of cytokine secretion enhancement, fibroblast proliferation, and collagen deposition [87]. In turn, synergistically acting flavonoids could increase fibroblast population and stimulate collagen production [88], while catechin could inhibit the degradation of the extracellular matrix by lowering the activity of elastase and collagenase and the regulation of matrix metalloproteinases through activation of NF-kB and AP-1 pathways [89].

### 3.7. Antioxidant Activity

#### 3.7.1. Free Radical-Scavenging Capacity

The results of DPPH and ABTS assays are presented in Table 1. The data indicated that the phenolic extract exhibited a significantly higher antioxidant capacity compared to the polysaccharide extract. Specifically, the DPPH IC_50_ value was 260 times lower for the phenolic extract (2.50 mg/mL) than that of the polysaccharide extract (660.92 mg/mL), indicating superior capacity to scavenge free DPPH radicals. In addition, the phenolic extract had a value of 16.47 mM TE/g for free ABTS scavenging compared to 1.25 mM TE/g for the polysaccharide extract.

These data were in accordance with a previous study on *Aloe vera* leaves, which reported higher antioxidant activity of the methanol extract (34.8–65.4%) compared to that of the water extract (15.4–48.1%), determined as the free DPPH radical scavenging activity at concentrations between 0.3 and 0.7 mg/mL [90]. Additionally, the DPPH IC_50_ values ranged between 0.03 mg/mL for the ethanol extract of *Aloe vera* leaves [91], 0.4 mg/mL for the ethanol extract of *Aloe vera* gel [52], and 1 mg/mL for the water extract of *Aloe vera* gel [92].

Similar variability was found in the literature for the results of ABTS assays applied to aloe extracts from different parts of the plant. Thus, as little as 0.001 mM TE/g of *Aloe vera* ethanol leaf extract obtained by UAE was reported [55], but 0.43 mM TE/g for the methanol extract of *Aloe vera* gel [93], 1.68 mM TE/g for the methanol extract of *Aloe vera* peel, and 46 mM TE/g for the methanol extract of *Aloe vera* leaves [94], as well as up to 1080 mM TE/g for the ethanol extract and 2600 mM TE/g for the methanol extract of *Aloe vera* gel, were also found [95]. Few studies have investigated the water extracts of *Aloe vera,* showing 0.43 mM TE/g in the case of the gel [93] and 0.98 mM TE/g in the case of the peel [94], values below those found in the present study for the water UAE extract of *Aloe vera* gel.

The wide range of antioxidant values across the cited studies confirms the variable composition of aloe plant according to the developmental stage and environmental conditions, and also that of aloe extracts according to the source and the applied extraction technique. Furthermore, it could also indicate the lack of standardization of DPPH and ABTS assays. However, all of these studies reveal that the phenolic compounds present in alcoholic extracts of *Aloe vera* gel represent good natural antioxidant agents with a high capacity to scavenge free radicals. The density functional theory was used to determine that aloesone is the best electron donor and, thus, the best antioxidant agent from aloe anthraquinones [96]. Also, chlorogenic acid and catechin were reported as major compounds of *Aloe vera* gel extract with good capacity to scavenge free DPPH radicals due to their high number of hydroxyl groups, and encapsulation in chitosan nanoparticles could enhance this valuable property [63].

#### 3.7.2. Effect on Intracellular ROS Production

**Figure 7:**
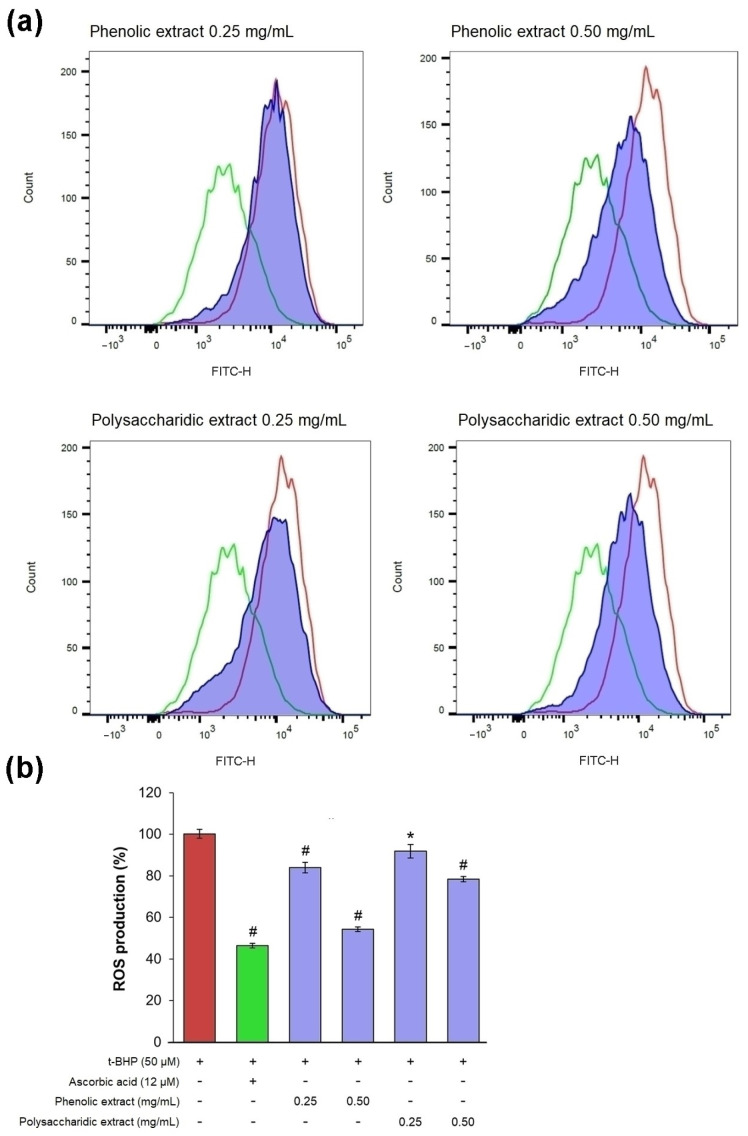
(**a**) Flow cytometry histograms showing count vs. fluorescence intensity in L929 fibroblasts after 48 h of treatment with phenolic and polysaccharide UAE extracts of *Aloe vera* gel (red—t-BHP treated cells, green—ascorbic acid treated cells, purple - aloe extracts treated cells). (**b**) Percentage of intracellular ROS production. * *p* < 0.05, # *p* < 0.01.

In an experimental model of oxidative stress in vitro, t-BHP treatment induced ROS production in excess, as confirmed by a pronounced shift to the right of the fluorescence peak, compared to the untreated culture (Figure 7a).

In cells treated with 0.25 mg/mL phenolic and polysaccharide extracts, the fluorescence signal was slightly shifted to the left compared to that of t-BHP-treated culture, while 0.5 mg/mL treatment significantly reduced the fluorescence intensity (Figure 7a). The results obtained in treated cells showed that the phenolic extract exhibited the highest antioxidant activity, with intracellular ROS production decreasing by 16% at 0.25 mg/mL and by 46% at 0.5 mg/mL (Figure 7b). It was observed that the value of ROS production in cells treated with 0.5 mg/mL phenolic extract was close to that in cells treated with ascorbic acid (47%), a known antioxidant agent. The polysaccharide extract showed antioxidant activity at a lower extent, decreasing intracellular ROS production by 8% at 0.25 mg/mL and by 22% at 0.5 mg/mL.

Ceravolo et al. [97] reported that 100 μg/mL *Aloe vera* extract exhibited protective activity against H_2_O_2_, significantly suppressing intracellular ROS production and inhibiting the MDA level, a marker of oxidative stress. Pretreatment with aloesone and aloin, as major metabolic compounds of *Aloe vera,* reduced LPS-induced ROS production in RAW264.7 cells, confirming they were the compounds responsible for the antioxidant activity of *Aloe vera* [66,67,68]. They could modulate oxidative stress at the cellular level through activation of the Nrf2 and inhibition of the MAPK and NF-kB signaling pathways, leading to fibroblast metabolism restoration [88].

### 3.8. Antibacterial Activity

**Figure 8:**
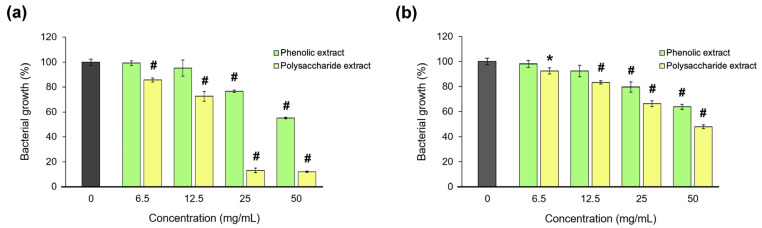
Bacterial growth of *S. aureus* (**a**) and *P. aeruginosa* (**b**) in the presence of the phenolic and polysaccharide UAE extracts of *Aloe vera* gel. * *p* < 0.05, # *p* < 0.01.

The antibacterial activity of *Aloe vera* extracts was tested against *S. aureus* and *P. aeruginosa*, as the most common pathogens associated with wound infections [98]. The microdilution test results are presented in Figure 8. The data showed that the polysaccharide extract was more potent than the phenolic extract in bacterial growth inhibition, and only on the Gram-positive strain. Thus, a bacteriostatic effect of the polysaccharide extract was found at an MIC of 25 mg/mL, and a bactericidal effect at an MBC of 50 mg/mL in *S. aureus* culture. In *P. aeruginosa* culture, the polysaccharide extract inhibited the bacterial growth by 50%, and the phenolic extract inhibited it by 35%.

The results were in accordance with previous studies showing the antibacterial activity of water extract from *Aloe vera* leaves to be 3.5 times higher than that of the ethanol extract against *S. aureus* [99]. No antibacterial activity against *S. aureus* was reported for the ethanol extract of *Aloe vera* gel, while the acetoacetate extract presented an MIC of 50 mg/mL and an MBC of 100 mg/mL [100]. In addition, *Aloe vera* gel revealed greater bactericidal efficacy against the Gram-positive *S. aureus* strain compared to the Gram-negative *P. aeruginosa*, with MBC values of 50 and 100 mg/mL, respectively [101]. Acemannan, a polymer present in aloe and composed of acetylated mannose units, was reported as an antibacterial agent and exerted 98% inhibition of *S. aureus* growth only when the extraction method maintained its high degree of acetylation [102], which probably enhanced the action of disrupting the plasma membrane and bacterial cell wall [49]. Further investigation focused on identifying the molecules responsible for the antibacterial activity of the polysaccharide extract of Aloe vera gel, which could significantly advance the development of novel therapeutic strategies for combating infections, potentially serving as alternatives to traditional antibiotics. These bioactive compounds might be integrated into various biomaterials to confer antibacterial properties or could be effectively delivered via nanomaterials, which serve as vehicles for targeted therapy [103].

The main biological activities demonstrated in this study for the phenolic and polysaccharide extracts of Aloe vera gel obtained by UAE using several biomimetic experimental models in vitro, which were developed for each phase of the wound-healing process, are summarized in Table 4.

All these results demonstrated that the phenolic extract of *Aloe vera* gel could exert major positive involvement and might be more beneficial in formulations intended for the initial phases of the wound-healing process, such as the inflammatory and proliferative stages, whereas the polysaccharide extract could play an important role and might be more suitable for use during later phases, such as the remodeling stage. Moreover, the phenolic extract displayed significant antioxidant activity, while the polysaccharide extract exerted antibacterial activity, suggesting that their combination with suitable biomaterials could enhance their efficacy in novel treatments of chronic wounds. In addition to their direct use or incorporation into pharmacological formulations, the extracts could be utilized for coating advanced biomaterials, such as electronic skin (e-skin). This application offers several advantages, including the capability to monitor physiological parameters such as blood pressure, pulse, and temperature through the properties of e-skin [104] while also ensuring good biocompatibility and supporting tissue regeneration.

## 4. Conclusions

A high yield of UAE of phenolic (44%) and polysaccharide (52%) compounds from lyophilized *Aloe vera* gel was established. The chemical characterization of the phenolic extract indicated high contents of aloin and ferulic, caffeic, and chlorogenic acid, next to quercetin, rutin, and myricetin. The polysaccharide extract was abundant mannose. Cell culture testing of the use of *Aloe vera* phenolic extract in experimental in vitro biomimetic models for the various phases of the wound-healing process indicated up to 28% TNF-α inhibition and 11% IL-8 inhibition at a concentration of 0.25 mg/mL, as well as stimulation of fibroblast proliferation by 18%, migration by 20%, and enhancement of collagen type I synthesis by 18% at a concentration of 0.5 mg/mL. Furthermore, the phenolic extract provided extra benefits through its significant antioxidant properties, which were exerted through free radical scavenging (2.50 mg/mL DPPH IC_50_ value, 16.47 mM TE/g) and 46% inhibition of intracellular ROS production. The polysaccharide extract was better in the remodeling phase, demonstrating a 25% increase in collagen synthesis at a concentration of 0.5 mg/mL and antibacterial activity against the Gram-positive *S. aureus* strain, with a MIC value of 25 mg/mL.

As a result, the practical implications of this study refer to a tailored approach, taking into consideration different effects of phenolic and polysaccharide extracts of Aloe vera gel for treatment adjustment in each specific phase of wound healing to accelerate the process and enhance chronic wound healing. Future work will envisage their use in novel, customizable formulations of skin dressing, either alone or in combination with other biomaterials, and preclinical tests will be conducted to effectively manage a rapid transition from the inflammatory phase to the final phases of the healing process.

## Figures and Tables

**Table 1:** Extraction yield, total phenolic content (TPC), total flavonoid content (TFC), total carbohydrate content (TCC), and free radical scavenging capacity of ultrasound-assisted extracts of phenolics and polysaccharides from *Aloe vera* gel.

	Yield (%)	TPC * (mg GAE/g)	TFC * (mg QE/g)	TCC * (mg GE/g)	DPPH IC_50_ (mg/mL)	ABTS * (mM TE/g)
Phenolic extract	44.02 ± 0.22	33.25 ± 1.71	1.96 ± 0.01	644.65 ± 10.46	2.50 ± 0.12	16.47 ± 0.01
Polysaccharide extract	52.14 ± 0.18	0.83 ± 0.11	ND	936.95 ± 6.94	660.92 ± 19.58	1.25 ± 0.17

* GAE is gallic acid equivalents, QE is quercetin equivalents, GE is glucose equivalents, TE is Trolox equivalents, DPPH is 2,2-Diphenyl-1-picrylhydrazyl test, and ABTS is 2,2′-azino-bis(3-ethylbenzothiazoline-6-sulfonic acid test.

**Table 2:** HPLC quantification of polyphenols identified in *Aloe vera* extract obtained by UAE in ethanol.

No.	Phenolic Compound	Retention Time (min)	Wavelength (nm)	mg/g Dry Extract
1	Aloin B	6.728	357	3.777 ± 0.37
2	Aloin A	7.540	357	5.158 ± 0.32
3	Chlorogenic acid	19.694	320	0.285 ± 0.02
4	Catechin hydrate	20.391	280	ND
5	Caffeic acid	21.550	320	0.316 ± 0.01
6	Syringic acid	22.269	280	ND
7	Rutin trihydrate	30.167	320	0.308 ± 0.01
8	Ferulic acid	31.177	320	0.328 ± 0.03
9	Apigenin-7-glucoside	31.586	320	0.199 ± 0.02
10	Quercetin 3-β-glucoside	32.753	320	ND
11	Kaempferol 3-β-D-glucopyranoside	35.936	320	0.222 ± 0.01
12	Myricetin	38.400	320	0.298 ± 0.01
13	Rosmarinic acid	39.407	320	ND
14	Quercetin dihydrate	43.908	320	0.351 ± 0.02
15	Apigenin	49.434	320	ND
16	Kaempferol	51.359	320	ND

ND is Not Detected.

**Table 3:** Quantification of monosaccharides identified in the hydrolysate of *Aloe vera* extract obtained by UAE in water.

No.	Monosaccharide Compound	Retention Time (min)	Wavelength (nm)	mg/g Dry Extract
1	Xylose	17.657	245	ND
2	Arabinose	18.083	245	ND
3	Glucose	18.150	245	313.881 ± 5.56
4	Ribose	18.324	245	ND
5	Rhamnose	18.550	245	ND
6	Mannose	19.330	245	534.273 ± 3.12
7	Fucose	19.537	245	ND
8	Galactose	20.303	245	16.680 ± 1.47
9	Galacturonic acid	22.304	245	ND
10	Glucuronic acid	23.144	245	11.092 ± 1.54

ND is Not Detected.

**Table 4:** In vitro effects in biomimetic models of wound healing phases and antioxidant and antimicrobial activity of *Aloe vera* phenolic and polysaccharide extracts obtained by UAE.

	Phenolic Extract		Polysaccharide Extract	
	0.25 mg/mL	0.5 mg/mL	0.25 mg/mL	0.5 mg/mL
Inflammatory phase	++		+	
Inhibition of TNF-α	28%	-	17%	-
Inhibition of IL-8	11%	-	8%	-
Proliferation phase	++		+	
Stimulation of fibroblast proliferation	11%	18%	8%	9%
Fibroblast morphology	normal	normal	normal	normal
Fibroblast migration rate	75%	88%	70%	83%
Remodeling phase	+		++	
Stimulation of collagen type I synthesis	11%	18%	15%	25%
Antioxidant activity	++		+	
IC50 DPPH	2.50 mg/mL		660.92 mg/mL	
TEAC	16.47 mM TE/g		1.25 mM TE/g	
Inhibition of ROS production	16%	46%	8%	22%
Antimicrobial activity	+		++	
Bactericidal activity against *S. aureus*	no		yes	
Bactericidal activity against *P. aeruginosa*	no		no	

(-) no biological activity; (+) moderate biological activity; (++) increased biological activity.

## Data Availability

The data are available from authors upon request.
